# Brown Seaweed *Sargassum siliquosum* as an Intervention for Diet-Induced Obesity in Male Wistar Rats

**DOI:** 10.3390/nu13061754

**Published:** 2021-05-21

**Authors:** Ryan du Preez, Marie Magnusson, Marwan E. Majzoub, Torsten Thomas, Christina Praeger, Christopher R. K. Glasson, Sunil K. Panchal, Lindsay Brown

**Affiliations:** 1 Functional Foods Research Group, University of Southern Queensland, Toowoomba, QLD 4350, Australia; r.dupreez@cqu.edu.au (R.d.P.); S.Panchal@westernsydney.edu.au (S.K.P.); 2 School of Science, Environmental Research Institute, University of Waikato, Tauranga 3112, New Zealand; marie.magnusson@waikato.ac.nz (M.M.); christopher.glasson@waikato.ac.nz (C.R.K.G.); 3 Centre for Marine Science and Innovation, University of New South Wales, Sydney, NSW 2052, Australia; m.majzoub@unsw.edu.au (M.E.M.); t.thomas@unsw.edu.au (T.T.); 4 School of Biological, Earth and Environmental Sciences, University of New South Wales, Sydney, NSW 2052, Australia; 5 MACRO—The Centre for Macroalgal Resources and Biotechnology, College of Marine and Environmental Sciences, James Cook University, Townsville, QLD 4811, Australia; tine.praeger@jcu.edu.au; 6 School of Health and Wellbeing, University of Southern Queensland, Ipswich, QLD 4305, Australia

**Keywords:** *Sargassum siliquosum*, brown seaweed, fucoidans, alginates, gut microbiota, metabolic syndrome

## Abstract

The therapeutic potential of *Sargassum siliquosum* grown in Australian tropical waters was tested in a rat model of metabolic syndrome. Forty-eight male Wistar rats were divided into four groups of 12 rats and each group was fed a different diet for 16 weeks: corn starch diet (C); high-carbohydrate, high-fat diet (H) containing fructose, sucrose, saturated and *trans* fats; and C or H diets with 5% *S. siliquosum* mixed into the food from weeks 9 to 16 (CS and HS). Obesity, hypertension, dyslipidaemia, impaired glucose tolerance, fatty liver and left ventricular fibrosis developed in H rats. In HS rats, *S. siliquosum* decreased body weight (H, 547 ± 14; HS, 490 ± 16 g), fat mass (H, 248 ± 27; HS, 193 ± 19 g), abdominal fat deposition and liver fat vacuole size but did not reverse cardiovascular and liver effects. H rats showed marked changes in gut microbiota compared to C rats, while *S. siliquosum* supplementation increased gut microbiota belonging to the family *Muribaculaceae*. This selective increase in gut microbiota likely complements the prebiotic actions of the alginates. Thus, *S. siliquosum* may be a useful dietary additive to decrease abdominal and liver fat deposition.

## 1. Introduction

Seaweeds are now a major industry worldwide, including a rediscovery of regional seaweed cuisines [1], but there are risks involved with some edible seaweeds [2]. The brown seaweed, *Saccharina japonica*, accounts for more than one-third of total world production, with over 11 million tonnes in 2018 [3]. Brown seaweeds have many commercial uses, including as food for human consumption [4], improving growth and meat quality of livestock [5], as a substrate for bioethanol production [6], as biostimulants for agricultural use [7] and as cation exchangers for wastewater remediation [8]. Further, the management of metabolic syndrome may be improved by some brown seaweeds, including *Ascophyllum nodosum* and *Fucus vesiculosis* [9].

Brown seaweeds also include the *Sargassum* genus that inhabits the Atlantic, Pacific and Indian oceans, including temperate, subtropical and tropical habitats; its biogeography suggests an origin at around 6.7 million years ago [10]. *Sargassum* species, primarily *S. natans* and *S. fluitans*, produced more than 20 million metric tonnes of biomass extending from West Africa to the Gulf of Mexico in 2018 [11]. Further, massive mats of *S. horneri* have occurred since 2015 off the southeastern coast of Korea [12]. However, value-adding pathways have been proposed for these golden tide seaweeds [13]. *Sargassum* (*fusiforme*) is already a traditional and highly prized food in Asia, with commercial aquaculture production of 54,624 tonnes of *S. fusiforme* in 2017 at a value of USD 28.7 million [14]. Further, *Sargassum* species produce many compounds with a wide range of biological actions, including protection against heat, pollution, stress, decreased oxygen concentration and ultraviolet radiation [15].

Commercial *Sargassum* species such as *S. fusiforme* contain alginates, laminarins and fucoidans as the major polysaccharides [16]; the xanthophyll, fucoxanthin, gives the brown seaweeds their colour. The species tested in this project, *S. siliquosum*, is found predominantly in the Sargasso Sea (western North Atlantic Ocean) and the Coral Sea (off the northeast coast of Australia) [17].

Functional foods are claimed to ameliorate metabolic syndrome [18], defined as a clustering of conditions that increase the risk of developing cardiovascular disease and type 2 diabetes; these factors include obesity, dyslipidaemia, hypertension, impaired glucose tolerance and fatty liver [19]. However, the effectiveness of *Sargassum* as a functional food has not been defined, although *Sargassum* products have shown effectiveness against metabolic diseases [20]. *Sargassum* species could exhibit antioxidant responses such as nitric oxide scavenging [21] and anti-inflammatory responses including reduced paw volumes in carrageenan-induced oedema [22]; both actions may be useful in metabolic syndrome. Further, fucoxanthin present in the whole seaweed may decrease obesity by modulating the expression of uncoupling protein-1; in a human trial, body weight was reduced by 5.5 kg following treatment with fucoxanthin at 2.4 mg/day for 16 weeks [23]. Fucoxanthin concentrations in brown seaweeds such as *Sphaerotrichia divaricata* [24] may be sufficient to improve physiological parameters when added to foods [25]. However, no studies have investigated *Sargassum* species on the range of pathophysiological changes that characterise metabolic syndrome.

We have reported that sulphated polysaccharides from the red seaweed *Sarconema filiforme* [26] and the green seaweed *Caulerpa lentillifera* [27] reversed the cardiovascular, metabolic and liver changes induced by a high-carbohydrate, high-fat diet in rats. Our hypothesis for this project was that the brown seaweed, *S. siliquosum*, as a source of non-sulphated (alginates and laminarins) and sulphated polysaccharides (fucoidans) and fucoxanthin, also reverses the signs of metabolic syndrome in this rat model [28]. This seaweed was chosen as it may be amenable to cultivation in tropical areas of Australia [29]. To test our hypothesis, we measured the parameters related to metabolic syndrome following chronic dietary intervention with *S. siliquosum*, including the structure and function of heart and liver, plasma biochemistry, glucose and insulin responses and body composition. Further, we characterised the changes in the composition of gut microbiota after seaweed treatment, as functional foods have the potential to reverse the changes induced in obesity [30,31]. The major mechanisms for possible responses to *S. siliquosum* could include the prebiotic actions of polysaccharides including fucoidans and alginates in the colon by reducing the intestinal absorption of carbohydrates and fats and also changing the gut microbiota, in combination with the anti-inflammatory effects of fucoxanthin [25]. The combination of these polysaccharides with fucoxanthin in the dried seaweed may also suppress the infiltration of inflammatory cells into the heart and liver.

## 2. Materials and Methods

### 2.1. Sargassum siliquosum Biomass Collection

**Figure 1:**
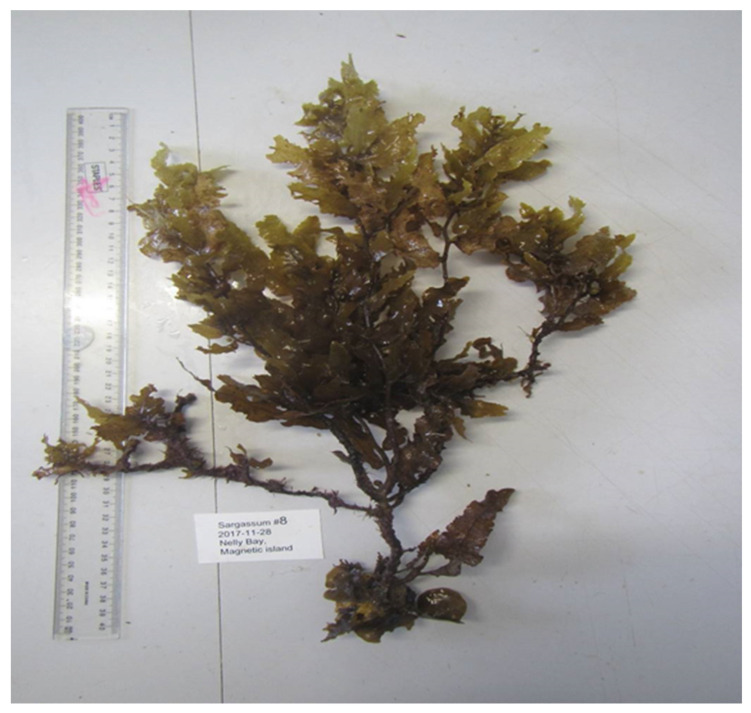
*Sargassum siliquosum* specimen with intact holdfasts and fresh new growth.

*S. siliquosum* was collected by snorkel from a rocky reef at approximately 2 m depth in Nelly Bay, Magnetic Island, QLD, Australia (19.1708° S, 146.8471° E), on 28 November 2017, and immediately transported to James Cook University, Townsville, QLD, Australia. Twelve specimens with intact holdfasts and fresh new growth were photographed (Figure 1) and selected for genetic barcoding. The remaining fresh biomass was briefly rinsed in batches in filtered seawater, followed by freshwater to remove sand, debris, invertebrates and epiphytes. The rinsed biomass was dried at 60 °C for 48 h, sorted to remove remaining foreign matter such as coral rubble in holdfasts, milled to 1 mm and homogenised. The biomass was stored with silica desiccant in vacuum-sealed bags at 4 °C until processing for experiments.

### 2.2. Genetic Barcoding

One blade from each of the twelve specimens selected for molecular barcoding was excised and cleaned in autoclaved seawater, using a soft paintbrush to remove debris and epiphytes. Each blade was dried to remove excess water and subsequently kept in individually labelled zip-lock bags with 50 g of silica beads to desiccate. Dried samples were sent to Victoria University of Wellington, New Zealand, for DNA extraction using a modified CTAB protocol [32]. The molecular marker mitochondrial gene cytochrome oxidase I (COI) was used to obtain sequences to assign *Sargassum* specimens to a genetic species group. The forward primer GazF2 (5′CCAACCAYAAAGATATWGGTAC3′) and reverse primer GazR2 (5′GGATGACCAAARAACCAAAA3′) were used [33]. Maximum likelihood (ML) phylogenetic trees were constructed in Iqtree with sequences downloaded from Genbank.

### 2.3. Compositional Analyses

All analyses were performed on five subsamples (*n =* 5) of milled and homogenised material, except for amino acid and constituent sugar analyses (*n =* 3) and fibre analysis (*n =* 2). For sugar analysis, hydrolysis of the biomass (10 mg) was performed in a two-step process: step 1: hydrolysis in 300 µL of 72% sulphuric acid at 20 °C for 1 h; step 2: hydrolysis in 3.6 mL of 1M sulphuric acid at 100 °C for 2 h [34]. Sugar hydrolysates were derivatised with 1-phenyl-3-methyl-5-pyrazolone (PMP) by a modified procedure [35]. Hydrolysates (40 µL) were neutralised with either 80 µL 1M NaOH prior to the addition of 400 µL of PMP-derivatising reagent (250 mM PMP and 400 mM ammonia in type 1 water) or 40 µL of 2-deoxy-glucose solution (10 mg/mL in type 1 water). This mixture was then heated on a magnetic stirrer at 70⁰C for 90 min. Derivatised samples were neutralised by addition of 400 µL of 0.8M formic acid followed by extraction of unbound PMP with 750 µL of chloroform. The aqueous layer was isolated and clarified by centrifugation (13,000 rpm for 5 min; Heraeus Pico 17 Thermo Scientific Centrifuge) prior to HPLC analysis. HPLC analysis was carried out with a Shimadzu LC-20AD Prominence fitted with a Restek Raptor C18 column (5 µm particle size, 150 mm × 4.6 mm; Cat # 9314565) with a flow rate of 0.8 mL/min and oven temperature of 40 ⁰C. Derivatised sugar standards or sample hydrolysates (5 µL) were injected and eluted with solvent A (0.1 M phosphate buffer at pH 7 in 10% acetonitrile) and solvent B (0.1 M phosphate buffer at pH 7 in 17% acetonitrile) with the following gradient programme: 25% B 0–15 min, 25–100% B at 15–40 min, 100% B 40–55 min, 25% B 55–60 min. Constituent sugars were quantified using PMP-derivatised calibration standards (0–10 mg/L) of fucose, galactose, glucose, mannose, rhamnose, xylose, mannuronic acid, guluronic acid, glucuronic acid and galacturonic acid. Proximate and elemental composition, trace elements, metals and metalloids, soluble and insoluble fibre were analysed as previously described [36]. Total lipids were quantified gravimetrically following extraction (60 °C, 1 h) of 200 mg (±0.1 mg) biomass in 5 mL chloroform:methanol (2:1, *v*:*v*). The extract was filtered, washed with 0.9% NaCl (*w*:*v*), allowed to separate into two phases, and the lower (organic) phase was collected for evaporation under nitrogen and subsequently weighed to give the total lipid content [37]. Fatty acids were simultaneously extracted and transesterified by heating (100 °C, 1 h) 50 mg (±0.1 mg) biomass in 2 mL methylation reagent (methanol, acetyl chloride, 20:1 (*v*/*v*)) with 300 µL nonadecanoic acid (C_19_H_38_O_2_; >99%, Sigma-Aldrich, Castle Hill, NSW, Australia) as internal standard. After cooling, 1 mL of hexane and 2 mL of deionised water were added, and the upper (hexane) phase was collected for analysis by GC–MS [37]. Total xanthophyll content was determined using ultraviolet–visible spectral peaks identified as xanthophyll carotenoids based on common and characteristic spectra. Fucoxanthin content was analysed using HPLC (diode-array detection at 450 nm) at Southern Cross University, Lismore, NSW, Australia.

### 2.4. Rats and Diets

Male Wistar rats (8–9 weeks old; 336 ± 2 g, *n =* 48) were sourced and housed as in previous studies [26,27] before being randomly divided into four groups (12 rats per group). Two groups received either corn starch or high-carbohydrate, high-fat diets (C and H, respectively) [28] for the full 16 weeks. The remaining groups received C and H diets for the first eight weeks and then received C or H diet with 5% *S. siliquosum* for the final eight weeks (CS and HS, respectively). Detailed composition of diets has been described in our published study [28].

### 2.5. Rat Measurements

**Figure 2:**
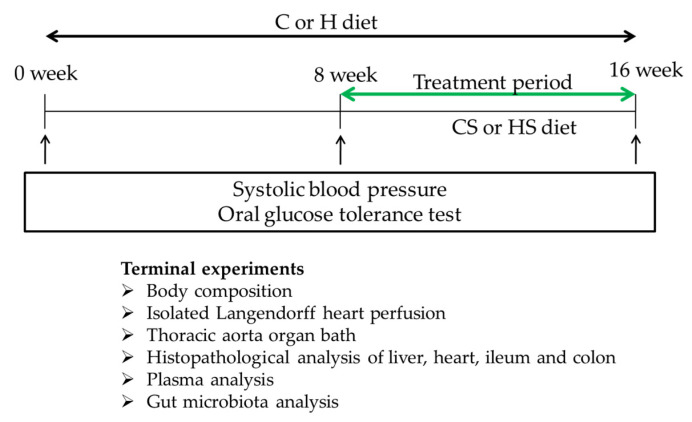
Study design to identify effects of *Sargassum siliquosum* intervention. C, rats fed with corn starch diet; CS, rats fed with corn starch diet +5% *Sargassum siliquosum*; H, rats fed with high-carbohydrate, high-fat diet; HS, rats fed with high-carbohydrate, high-fat diet +5% *Sargassum siliquosum*.

The interventions and measured parameters are given in Figure 2. Rats were anaesthetised using isoflurane for measurements of body composition using dual-energy X-ray absorptiometry, systolic blood pressure and abdominal circumference measurement [26]. Oral glucose and insulin tolerance tests and indirect calorimetry were performed as previously described [28,38]. Following euthanasia, heparin was injected before blood collection, centrifugation, plasma isolation and analysis and then diastolic stillness measurements, organ weights, thoracic aortic responses and histological analyses [28].

Post euthanasia, two or three faecal pellets were collected from the colon of each rat and processed as described previously to obtain gut microbiota composition [26,27]. Data were presented and analysed for statistical significance as detailed in previous studies [26,27].

## 3. Results

### 3.1. Sargassum siliquosum Identification and Compositional Analyses

**Figure 3:**
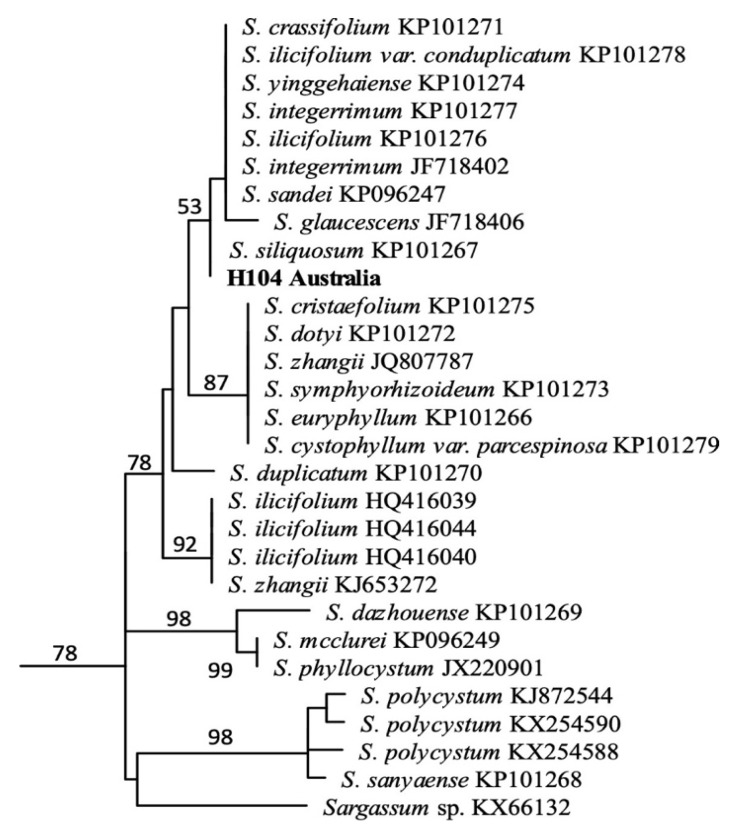
Genetic barcoding of *Sargassum* species: maximum likelihood tree of cytochrome oxidase I marker sequence data. Numbers near each node refer to bootstrap support values. Numbers accompanying the species names are GenBank accession numbers for the sequences used in the analysis. The specimens collected here are referred to as H104 Australia.

**Figure 4:**
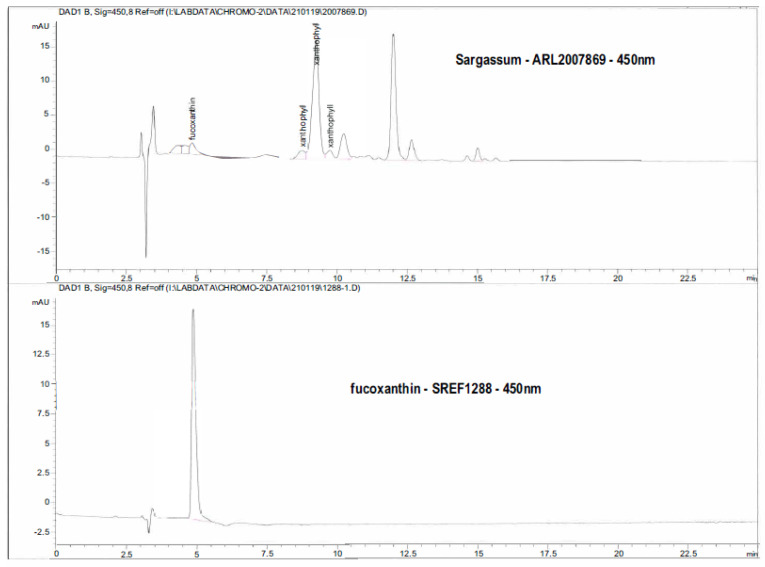
Chromatograms using diode-array detection to estimate xanthophyll (total) and fucoxanthin concentrations in *Sargassum siliquosum*.

DNA barcoding using the cytochrome oxidase subunit 1 (COI) gene was used for identification of the *Sargassum* samples collected. Sequences could be generated for 9 (out of 10) specimens and all sequences were identical. A phylogenetic tree was compiled from comparison with sequences downloaded from Genbank. A trimmed phylogenetic tree (Figure 3; full tree in Supplementary Figure S1) shows a clustering, with moderate bootstrap support (53%), of similar *Sargassum* species; these Genbank accessions include several named *Sargassum* species (*S. ilicifolium*, *S. integerrimum*) including *S. siliquosum*. The COI sequences of this grouping were identical, except for our samples and *S. siliquosum*, which shared a single synapomorphy. Based on these data and distributional data, we identified this species as *S. siliquosum*. These samples of *S. siliquosum* contained (in % of dry weight) 57.8% carbohydrate (11 mol% fucose, 24.4 mol% glucose, 26.4 mol% mannuronic acid and 24.2 mol% guluronic acid), 1.7% lipid, 4.02% protein, 41.4% dietary fibre including 33.3% as soluble fibre with 7.5% K, 1.9% Ca, 1.2% Na and 0.97% S as the major elements (Table 1 and Table 2; Supplementary Tables S1 and S2). Hydrolysates of *Sargassum* biomass indicated a total of 9.47% *w*/*w* sugars, with high contents of mannuronic acid and guluronic acid and glucose, consistent with the presence of alginate and storage carbohydrates/cellulose, respectively. Fucose was present at 0.91% *w*/*w*, consistent with the presence of fucoidan at ≥1.5% *w*/*w* as the monosulphated sodiated salt (Table 1). In *S. siliquosum*, glutamic acid and aspartic acid were the most common amino acids (Supplementary Table S1) and potassium and calcium the most common elements (Supplementary Table S2). Total xanthophyll content was 0.00746% *w*/*w* and fucoxanthin content was 0.00058% *w*/*w* based on HPLC data (Figure 4).

### 3.2. Physiological Parameters

**Figure 5:**
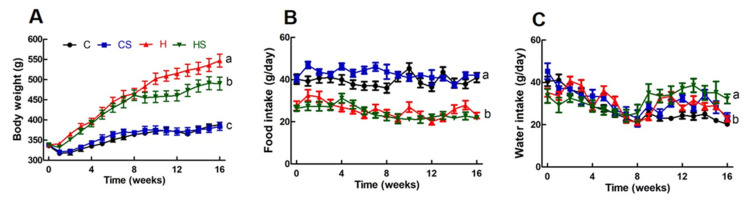
Effects of *S. siliquosum* on (**A**) body weight, (**B**) food intake and (**C**) water intake. Means with unlike superscripts (a, b or c) differ, *p* < 0.05. C, rats fed with corn starch diet; CS, rats fed with corn starch diet + 5% *Sargassum siliquosum*; H, rats fed with high-carbohydrate, high-fat diet; HS, rats fed with high-carbohydrate, high-fat diet + 5% *Sargassum siliquosum*.

Metabolic changes were more marked than cardiovascular or liver changes following intervention with *S. siliquosum*. During weeks 9–16, the food consumption was unchanged in C and CS, and in H and HS (Table 3; Figure 5). CS and HS rats drank more water than their respective controls, C and H (Table 3, Figure 5). During weeks 9–16, there was no difference in energy intake between C and CS rats or H and HS rats. After sixteen weeks of feeding, the body weight of H rats was higher than C rats; HS rats had lower body weights than H rats. There was no difference in lean mass across all groups. Fat mass was higher in H rats compared to C rats, which were similar to CS rats. HS rats had decreased retroperitoneal, liver and whole-body fat compared to H rats (Table 3). In HS rats, there were no changes in lipid profile or glucose and insulin metabolism compared to H rats. *S. siliquosum* did not affect the plasma triglyceride concentrations in HS and CS rats compared to H and C rats, respectively, whereas plasma total cholesterol concentrations were unaffected by diet or treatment (Table 3). H diet increased basal blood glucose concentrations compared to C diet but *S. siliquosum* did not change basal blood glucose concentrations or blood glucose area under the curve (Table 3).

**Figure 6:**
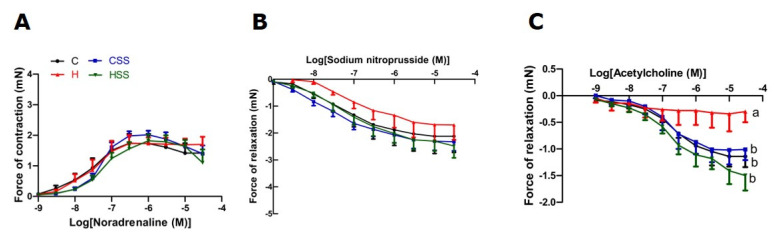
Effects of *S. siliquosum* on thoracic aortic responses to (**A**) noradrenaline, (**B**) sodium nitroprusside and (**C**) acetylcholine. Means with unlike superscripts (a or b) differ, *p* < 0.05. C, rats fed with corn starch diet; CS, rats fed with corn starch diet + *Sargassum siliquosum*; H, rats fed with high-carbohydrate, high-fat diet; HS, rats fed with high-carbohydrate, high-fat diet + 5% *Sargassum siliquosum* (HS).

**Figure 7:**
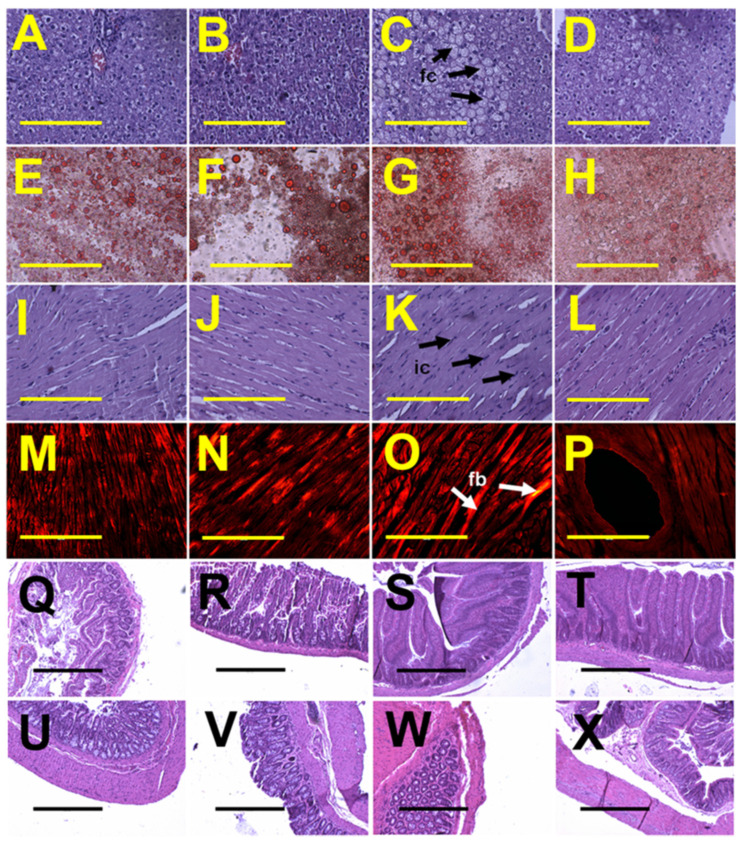
Histological analysis of liver, heart, ileum and colon. (**A**–**D**) showing haematoxylin and eosin staining and (**E**–**H**) showing oil red O staining to identify liver fat deposition; (**I**–**L**) showing haematoxylin and eosin staining to identify heart inflammation; (**M**–**P**) showing picrosirius red staining to identify heart fibrosis; (**Q**–**T**) showing haematoxylin and eosin stain of ileum and (**U**–**X**) showing haematoxylin and eosin stain of colon in rats fed with corn starch diet (**A**,**E**,**I**,**M**,**Q**,**U**), rats fed with corn starch diet + *Sargassum siliquosum* (**B**,**F**,**J**,**N**,**R**,**V**), rats fed with high-carbohydrate, high-fat diet (**C**,**G**,**K**,**O**,**S**,**W**) and rats fed with high-carbohydrate, high-fat diet + *Sargassum siliquosum* (**D**,**H**,**L**,**P**,**T**,**X**). Fat cells = fc; inflammatory cells = ic; fibrosis = fb. Scale bar is 200 µm for (**A**–**P**) (20×) and 100 µm for (**Q**–**X**) (10×).

Systolic blood pressure of H and HS rats was higher than C and CS rats at 8 weeks (Table 3). Systolic blood pressure and left ventricular diastolic stiffness in H rats were higher than in C rats at 16 weeks. Intervention with *S. siliquosum* did not alter systolic blood pressure or left ventricular stiffness (Table 3). Left ventricular wet weights with septum and right ventricular wet weights were not different among the groups (Table 3). Intervention with *S. siliquosum* did not alter noradrenaline-induced contraction or sodium nitroprusside-induced relaxation responses of thoracic aorta, while acetylcholine-induced relaxation was improved in HS rats compared to H rats (Figure 6). Left ventricles from H rats showed infiltration of inflammatory cells and collagen deposition, whereas these changes were absent in left ventricles from C rats; intervention with *S. siliquosum* did not alter collagen deposition (Figure 7). Fat vacuole area and infiltration of inflammatory cells were increased in livers from H rats compared to C rats; these parameters were reduced in HS rats (Figure 7). Plasma activities of ALT and AST were unchanged between groups (Table 3).

### 3.3. Gut Structure and Microbiota

The structures of the ileum and colon were unchanged by diet or intervention, defined by crypt depth, villi length and goblet cells, and lack of inflammatory cell infiltration (Figure 7).

**Figure 8:**
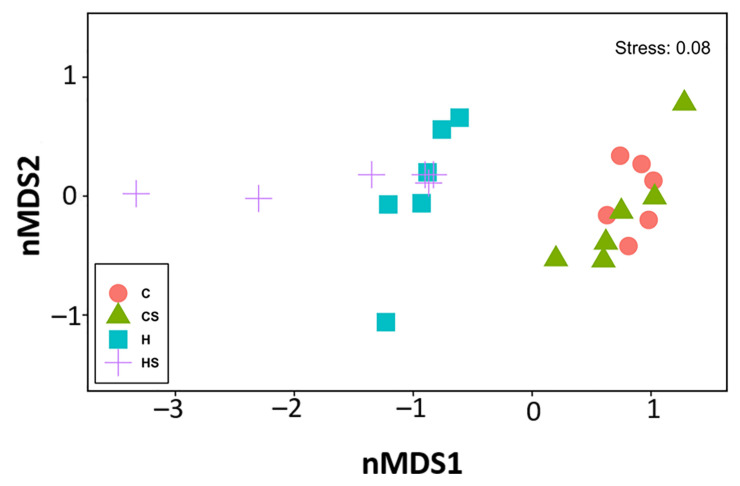
Multidimensional scaling plot for the Bray–Curtis dissimilarities of bacterial gut communities. C, rats fed with corn starch diet; CS, rats fed with corn starch diet + *Sargassum siliquosum*; H, rats fed with high-carbohydrate, high-fat diet; HS, high-carbohydrate, high-fat diet + *Sargassum siliquosum*.

For gut microbiota characterisation, a total of 799,215 quality-filtered sequences were clustered into 1307 zOTUs; Good’s coverage score of 99.69 ± 0.12% suggested full recovery of bacterial communities. Shannon’s diversity and richness indices were unchanged among the groups (Supplementary Figure S2). Diet and *S. siliquosum* affected the overall bacterial community structure individually as well as through their interaction (Figure 8; Supplementary Table S3).

**Figure 9:**
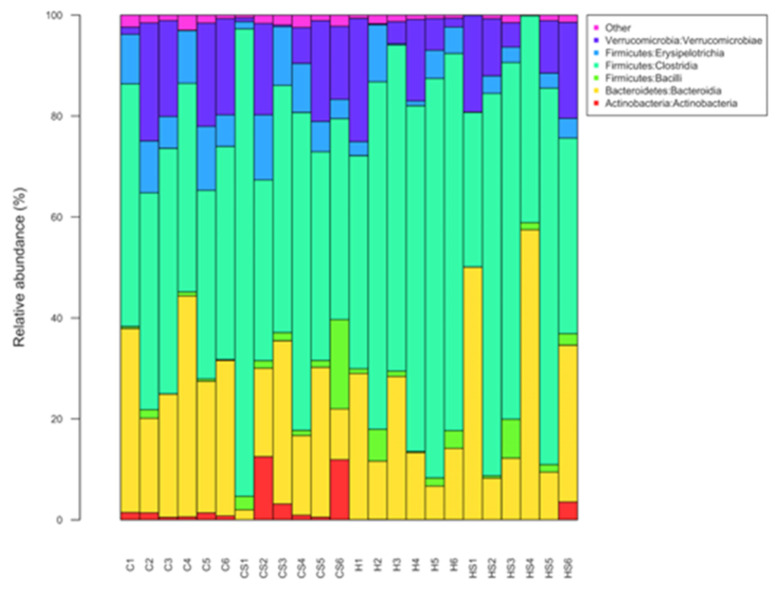
Taxonomic profiles of bacterial communities of all faecal samples shown at the class level. C, rats fed with corn starch diet; CS, rats fed with corn starch diet + *Sargassum siliquosum*; H, rats fed with high-carbohydrate, high-fat diet; HS, rats fed with high-carbohydrate, high-fat diet + *Sargassum siliquosum*.

Actinobacteria, Bacteroidia, Bacilli, Clostridia, Erysipelotrichia and Verrucomicrobia were the most abundant bacterial classes in the faecal samples (Figure 9). Coriobacteriia, Melainabacteria, Deferribacteres, Saccharimonadia, Alphaproteobacteria, Deltaproteobacteria, Gammaproteobacteria and Mollicutes were observed at lower abundance levels (<1%) in some faecal samples. A higher abundance of bacteria from the class Clostridia was observed for the H diet samples, and this was more pronounced for control groups (C: 43.45 ± 1.75%, H: 66.35 ± 5.26%, *p* = 0.0005) compared to groups supplemented with *Sargassum* (CS: 53.58 ± 8.73%, HS: 55.23 ± 8.40%) (Figure 9). Within the class Bacteroidia, there was an increase in the relative abundance of bacteria from the family *Muribaculaceae* in HS rats (24.34%) compared to H rats (10.35%) (*p* = 00063). Further information on the gut microbiota, including at genus level (Supplementary Figure S3), and changes due to diet intervention (Supplementary Tables S3–S6), are included in the Supplementary Information.

## 4. Discussion

Seaweeds containing complex mixtures of polysaccharides, peptides, pigments, minerals and omega-3 fatty acids have been shown to improve the signs of metabolic syndrome [39]. This study showed that 5% *S. siliquosum* supplementation in rats with diet-induced metabolic syndrome decreased body weight and decreased retroperitoneal fat and liver fat but had no effect on systolic blood pressure, liver enzyme activities, lipid profile or glucose and insulin metabolism. Similar responses have been shown with other *Sargassum* species. As examples, *S. thunbergii* decreased obesity, serum insulin, triglycerides and cholesterol in high-fat-diet-fed mice [40] and *S. polycystum* decreased damage to the liver in high-sugar, high-fat diet + streptozotocin-induced type 2 diabetic rats [41].

*Sargassum* species contain polysaccharides, predominantly (in decreasing content) alginates, fucoidans and laminarans; these are the major constituents of the high fibre content of *S. siliquosum* [42] as in other brown seaweeds [43]. Oral intake of alginates leads to the formation of alginate gels in the small intestine, proposed as the most likely mechanism for alginate-related slowed nutrient absorption leading to body weight loss [44]. In healthy humans, a dose of 9.9–15 g/day of sodium alginate increased satiety and reduced energy intake, suggesting that increased viscosity causing swelling of gastrointestinal contents [45]. In a human trial using energy restriction (~1250 kJ/day) and 15 g/day of alginates as a beverage for 12 weeks, body weight was reduced compared to the placebo group, mainly attributed to body fat reduction [46]. This human dose of 15 g alginates/day approximates to 3.1 g alginates/day in rats using the Reagan–Shaw scaling equation [47].

Fucoidan from *S. fusiforme* changed the gut microbiota, particularly by increasing the Bacteroidetes to Firmicutes ratio, to decrease streptozotocin-induced hyperglycaemia in mice [48]. Further, fucoidan from *S. fusiforme* decreased high-fat-diet-induced insulin resistance in mice by activating the Nrf2 pathway, changing the gut microbiota and reducing intestinal inflammation [49]. Fucoidan supplementation of 100 mg/kg/day in high-fat-diet-fed rats improved blood lipid concentrations, decreased fat deposition in the liver and changed the gut microbiota [50]. However, this fucoidan dose was around three times higher than the dose of around 34 mg/kg/day in the current study, so fucoidan is unlikely to be the major bioactive compound in *S. siliquosum*.

Inhibiting digestive enzymes including α-amylase, α-glucosidase, pepsin and lipase by seaweed polysaccharides and polyphenols may modulate obesity and cardiovascular risk [51]. In an in vitro study, *S. siliquosum* and *S. polycystum* inhibited angiotensin-converting enzyme, α-amylase and α-glucosidase [52]. The role of these mechanisms in the current study is unknown.

The biological responses could be caused by the xanthophyll, fucoxanthin. In high-fat-diet-induced obese rats, fucoxanthin at doses of 0.083 and 0.167 mg/kg reduced white adipose tissue weight, accumulation of hepatic lipid droplets and perirenal adipocyte size [53]. These doses are 6–12 times higher than the fucoxanthin intake in the current study, so it seems unlikely that the metabolic responses with *S. siliquosum* are primarily due to the actions of fucoxanthin. The metabolic actions of phenolic acids and other xanthophyll compounds present in this seaweed are unknown but they could be additive to responses produced by the alginates.

Polyunsaturated fatty acids such as EPA and DHA decreased abdominal obesity and total body fat in the same rat model of metabolic syndrome as the current study [54]. However, the dose of EPA or DHA in this study was approximately 1500 mg/kg/day, in contrast to the dose of total polyunsaturated fatty acids in the current study of around 11 mg/kg/day. Thus, the changes in metabolic parameters following intervention with *S. siliquosum* are unlikely to be due to an increased intake of polyunsaturated fatty acids.

Not unexpectedly, a diet with higher simple sugars and *trans* and saturated fats changed the gut microbiome, similar to results in our previous study [26]. Supplementation with *S. siliquosum* caused a further change in the gut microbiota, including an increase in bacteria belonging to the family *Muribaculaceae* (also known as S24-7). Members of this family are understudied but have recently been recognised for their versatile metabolism of complex carbohydrates [55,56] and their potential for increased succinate, acetate and propionate production [57]. Members of this family have also been linked to longevity in rodents [58] and, based on our results, could decrease fat absorption or deposition, as these parameters were reduced following intervention with *S. siliquosum*.

One potential mechanism would be that the polysaccharides from *Sargassum* act as prebiotics to alter the changed gut microbiota in obesity, as shown by other seaweeds [59]. Alginates may be degraded by specific bacteria, including members of the *Muribaculaceae*, thereby increasing concentrations of short-chain fatty acids, which could lead to human health benefits [60]. Unsaturated alginate polysaccharides have been reported to act as effective prebiotics by increasing beneficial gut bacteria and decreasing inflammogenic bacteria in mice with high-fat-diet-induced obesity [61]. An improvement in gut health was suggested by the in vitro fermentation of a polysaccharide from *S. thunbergia* by colonic microbiota to short-chain fatty acids [62].

The current study could be extended by including the measurement of obesity-related plasma and tissue biomarkers such as adiponectin and C-reactive protein, inflammatory mediators such as TNF and functional changes in the colon such as short-chain fatty acid production. These data could allow further interpretation of the mechanisms of action of this seaweed.

## 5. Conclusions

The tropical Australian brown seaweed, *S. siliquosum*, reduced liver and abdominal fat accumulation in high-carbohydrate high-fat fed rats. As this seaweed contains alginates as the major polysaccharide, these alginates may act as prebiotics in the intestine to increase concentrations of short-chain fatty acids. These actions are possibly complemented by an increase in specific bacteria such as *Muribaculaceae* in the gut microbiome. These intestinal changes could then lead to systemic anti-inflammatory effects in the heart and liver. We conclude that the tropical Australian seaweed, *S. siliquosum*, should now be included with other local tropical seaweeds such as *Sarconema filiforme* [26] and *Caulerpa lentillifera* [27] as dietary additives in clinical studies designed to measure improvements in the signs of metabolic syndrome.

## Figures and Tables

**Table 1:** Proximate composition (% of dry weight) of *Sargassum siliquosum*.

Lipid	Protein (Sum Amino Acids)	Ash	Moisture	Carbohydrate *	Dietary Fibre		
					Total	Soluble	Insoluble
1.7 ± 0.4	4.02 ± 0.1	27.7 ± 1.3	9.1 ± 0.5	57.8 ± 0.5	40.2, 42.6	5.36, 11.0	34.9, 31.6

* by difference. Values are presented as mean ± SEM, *n =* 3 for protein; and *n =* 2 for dietary fibre, both values are provided.

**Table 2:** Elemental composition (% of dry weight) of *Sargassum siliquosum*.

C	H	N	S	I
29.00 ± 0.33	4.31 ± 0.09	0.91 ± 0.02	1.18 ± 0.05	0.038 ± 0.002

Values are presented as mean ± SEM, *n =* 5.

**Table 3:** Responses to *Sargassum siliquosum*.

Variables	C	CS	H	HS	*p* Value		
					Diet	Treatment	Interaction
Physiological variables							
Body weight 0 weeks, g	337 ± 1	338 ± 1	339 ± 1	338 ± 1	0.38	1.00	0.38
Body weight 8 weeks, g	366 ± 7 ^b^	369 ± 5 ^b^	445 ± 10 ^a^	461 ± 13 ^a^	<0.0001	0.39	0.55
Body weight 16 weeks, g	388 ± 10 ^c^	384 ± 10 ^c^	547 ± 14 ^a^	490 ±16 ^b^	<0.0001	0.047	0.08
Lean mass 16 weeks, g	292 ± 15	282 ± 6	299 ± 12	281 ± 10	0.78	0.20	0.71
Fat mass 16 weeks, g	75 ± 15 ^c^	86 ± 8 ^c^	248 ± 27 ^a^	193 ± 19 ^b^	<0.0001	0.24	0.08
Food intake 0–8 weeks, g/day	43.2 ± 2.2 ^a^	44.2 ± 1.0 ^a^	26.6 ± 1.1 ^b^	26.4 ± 1.0 ^b^	<0.0001	0.76	0.65
Food intake 9–16 weeks, g/day	44.0 ± 1.2 ^a^	41.1 ± 0.9 ^a^	23.9 ± 0.9 ^b^	22.3 ± 0.6 ^b^	<0.0001	0.022	0.49
Xanthophylls intake (total), mg/kg/day	-	0.40 ± 0.01	-	0.17 ± 0.01	-	-	-
Fucoxanthin intake, mg/kg/day	-	0.031 ± 0.001	-	0.013 ± 0.001	-	-	-
Alginate intake, mg/kg/day	-	1764 ± 15	-	749 ± 9	-	-	-
Fucoidan intake, mg/kg/day	-	80.2 ± 0.8	-	34.1 ± 10.4	-	-	-
Iodine, mg/kg/day	-	41 ± 1	-	17 ± 1	-	-	-
Polyunsaturated fatty acid intake, mg/kg/day	-	26.2 ± 0.2	-	11.1 ± 0.1	-	-	-
Water intake 0–8 weeks, g/day	31.8 ± 1.6	31.8 ± 2.0	32.4 ± 1.4	29.1 ± 1.2	0.57	0.37	0.37
Water intake 9–16 weeks, g/day	21.7 ± 1.4 ^c^	28.5 ± 0.7 ^b^	28.8 ± 1.3 ^b^	34.8 ± 1.4 ^a^	<0.0001	<0.0001	0.76
Energy intake 0–8 weeks, kJ/day	485 ± 25 ^b^	496 ± 11 ^b^	607 ± 19 ^a^	584 ± 20 ^a^	<0.0001	0.76	0.39
Energy intake 9–16 weeks, kJ/day	470 ± 13 ^b^	457 ± 6 ^b^	536 ± 15 ^a^	534 ± 13 ^a^	<0.0001	0.55	0.66
Feed efficiency 9–16 weeks, g/kJ	0.05 ± 0.01 ^b^	0.03 ± 0.01 ^b^	0.19 ± 0.02 ^a^	0.05 ± 0.01 ^b^	0.0001	0.08	0.042
Abdominal circumference 16 weeks, cm	18.7 ± 0.5 ^b^	18.5 ± 0.2 ^b^	21.5 ± 0.2 ^a^	22.0 ± 0.5 ^a^	<0.0001	0.73	0.42
Body mass index, g/cm^2^	0.61 ± 0.03 ^b^	0.65 ± 0.01 ^b^	0.81 ± 0.02 ^a^	0.75 ± 0.02 ^a^	<0.0001	0.62	0.019
Retroperitoneal fat, mg/mm	210 ± 20 ^c^	218 ± 13 ^c^	673 ± 54 ^a^	495 ± 50 ^b^	<0.0001	0.052	0.034
Epididymal fat, mg/mm	89 ± 11 ^b^	65 ± 7 ^b^	250 ± 36 ^a^	115 ± 15 ^b^	<0.0001	<0.0001	0.003
Omental fat, mg/mm	139 ± 14 ^b^	165 ± 9 ^b^	325 ± 34 ^a^	272 ± 22 ^a^	<0.0001	0.53	0.07
Total abdominal fat, mg/mm	437 ± 42 ^c^	448 ± 25 ^c^	1107 ± 57 ^a^	907 ± 71 ^b^	<0.0001	0.12	0.08
Visceral adiposity, %	5.2 ± 0.5 ^b^	5.3 ± 0.2 ^b^	9.3 ± 1.1 ^a^	8.3 ± 0.6 ^a^	<0.0001	0.47	0.38
Liver wet weight, mg/mm	261 ± 11 ^b^	244 ± 10 ^b^	380 ± 12 ^a^	376 ± 14 ^a^	<0.0001	0.44	0.63
Cardiovascular variables							
**Systolic blood pressure 8 weeks, mmHg**	125 ± 4 ^b^	121 ± 2 ^b^	137 ± 3 ^a^	134 ± 3 ^a^	0.0003	0.27	0.87
Systolic blood pressure 16 weeks, mmHg	123 ± 2 ^b^	122 ± 2 ^b^	138 ± 3 ^a^	135 ± 4 ^a^	0.0003	0.57	0.77
Left ventricle + septum, mg/mm	22.9 ± 1.1	22.8 ± 0.7	25.2 ± 1.1	23.0 ± 0.7	0.17	0.20	0.25
Right ventricle, mg/mm	4.5 ± 0.7	3.9 ± 0.3	5.3 ± 0.2	5.3 ± 0.2	0.004	0.41	0.41
Left ventricular diastolic stiffness, κ	22.1 ± 0.8 ^b^	22.9 ± 0.7 ^b^	30.5 ± 1.2 ^a^	29.4 ± 1.3 ^a^	<0.0001	0.90	0.42
Left ventricular collagen area, %	10 ± 2 ^b^	11 ± 3 ^b^	33 ± 3 ^a^	29 ± 5 ^a^	0.0003	0.67	0.49
Metabolic variables							
Plasma total cholesterol, mmol/L	1.56 ± 0.08	1.68 ± 0.06	1.57 ± 0.10	1.86 ± 0.19	0.53	0.18	0.57
Plasma triglycerides, mmol/L	0.43 ± 0.02 ^b^	0.42 ± 0.04 ^b^	1.88 ± 0.31 ^a^	1.54 ± 0.28 ^a^	<0.0001	0.40	0.43
Alanine transaminase, U/L	34 ± 4	53 ± 6	38 ± 2	48 ± 6	0.94	0.028	0.48
Aspartate transaminase, U/L	116 ± 2	150 ± 19	120 ± 12	142 ± 20	0.92	0.18	0.77
Liver inflammatory cells, cells/200 µm^2^	6 ± 1 ^b^	7 ± 1 ^b^	25 ± 2 ^a^	26 ± 3 ^a^	<0.0001	0.62	1.00
Liver fat vacuoles area, fat vacuoles/200 µm^2^	13.1 ± 1.7 ^c^	15.6 ± 2.4 ^c^	88.6 ± 3.4 ^a^	55.2 ± 2.9 ^b^	<0.0001	<0.0001	<0.0001
Oral glucose tolerance test							
Basal blood glucose 0 weeks, mmol/L	2.6 ± 0.1	2.6 ± 0.1	2.6 ± 0.2	2.7 ± 0.1	0.70	0.70	0.70
Area under the curve 0 weeks, mmol/L × minute	632 ± 30	594 ± 20	606 ± 19	552 ± 12	0.11	0.033	0.70
Basal blood glucose 8 weeks, mmol/L	2.9 ± 0.2 ^b^	2.6 ± 0.1 ^b^	3.3 ± 0.1 ^a^	3.5 ± 0.1 ^a^	<0.0001	0.70	0.058
120-minute blood glucose 8 weeks, mmol/L	3.5 ± 0.2 ^b^	3.7 ± 0.1 ^b^	5.0 ± 0.1 ^a^	5.2 ± 0.2 ^a^	<0.0001	0.27	1.00
Area under the curve 8 weeks, mmol/L × minute	530 ± 15 ^b^	537 ± 9 ^b^	657 ± 22 ^a^	682 ± 15 ^a^	<0.0001	0.31	0.57
Basal blood glucose 16 weeks, mmol/L	2.8 ± 0.2	3.0 ± 0.2	3.3 ± 0.2	3.4 ± 0.1	0.55	0.84	0.95
120-minute blood glucose 16 weeks, mmol/L	3.9 ± 0.2 ^b^	3.7 ± 0.1 ^b^	4.8 ± 0.3 ^a^	4.5 ± 0.1 ^a^	<0.0001	0.13	0.76
Area under the curve 16 weeks, mmol/L × minute	501 ± 21 ^b^	523 ± 14 ^b^	617 ± 25 ^a^	604 ± 9 ^a^	<0.0001	0.79	0.29
Insulin tolerance test							
120-minute blood glucose 8 weeks, mmol/L	2.9 ± 0.4 ^b^	2.7 ± 0.2 ^b^	4.5 ± 0.3 ^a^	4.4 ± 0.2 ^a^	<0.0001	0.58	0.85
Area under the curve 8 weeks, mmol/L × minute	247 ± 58 ^b^	234 ± 32 ^b^	408 ± 21 ^a^	390 ± 18 ^a^	<0.0001	0.65	0.94
120-minute blood glucose 16 weeks, mmol/L	2.7 ± 0.3 ^b^	3.3 ± 0.2 ^b^	4.5 ± 0.4 ^a^	4.3 ± 0.2 ^a^	<0.0001	0.46	0.14
Area under the curve 16 weeks, mmol/L × minute	208 ± 37 ^b^	168 ± 27 ^b^	404 ± 54 ^a^	420 ± 28 ^a^	<0.0001	0.74	0.44

Values are presented as mean ± SEM, *n* = 10–12. Means in a row with unlike superscripts (a, b or c) differ, *p* < 0.05. C, rats fed with corn starch diet; CS, rats fed with corn starch diet + *Sargassum siliquosum*; H, rats fed with high-carbohydrate, high-fat diet; HS, rats fed with high-carbohydrate, high-fat diet + *Sargassum siliquosum*.

## Data Availability

The data presented in this study are available on request from the corresponding author.

## Supplementary Materials

The following are available online at https://www.mdpi.com/article/10.3390/nu13061754/s1, Figure S1: Genetic barcoding of *Sargassum* species: Maximum likelihood tree of cytochrome oxidase I marker sequence data (full phylogenetic tree); Figure S2: (A) Shannon diversity and (B) richness of faecal samples; Figure S3: Taxonomic profiles of bacterial communities of all faecal samples shown at the genus level; Table S1: Fatty acids and amino acids content (% of dry weight) in *Sargassum siliquosum*; Table S2: Metals and metalloids content (mg/kg of dry weight) in *Sargassum siliquosum*; Table S3: PERMANOVAs based on Bray–Curtis similarity measure for square-root transformed abundances; Table S4: Summarised differential zOTU abundance; Table S5: Effects of diet on relative abundance of zOTUs; Table S6: Effects of treatment on relative abundance of zOTUs.
