# Supplementary material for: Brown Seaweed Sargassum siliquosum as an Intervention for Diet-Induced Obesity in Male Wistar Rats
Source: Nutrients. 2021 May 21;13(6):1754. doi: 10.3390/nu13061754 (PMC8224310; doi:10.3390/nu13061754)
Supplement: Supplementary file 1 [file nutrients-13-01754-s001.zip › nutrients-1192513-supplementary.pdf]

nutrients

Article

Brown Seaweed *Sargassum siliquosum* as an Intervention for Diet-Induced Obesity in Male Wistar Rats

Ryan du Preez [1,†], Marie Magnusson [2], Marwan E. Majzoub [3,4], Torsten Thomas [3,4], Christina Praeger [5], Christopher R. K. Glasson [2], Sunil K. Panchal [1,‡], Lindsay Brown [1,6,*]

[1] Functional Foods Research Group, University of Southern Queensland, Toowoomba, QLD 4350, Australia; r.dupreez@cqu.edu.au (R.d.P.); S.Panchal@westernsydney.edu.au (S.K.P.)
[2] School of Science, Environmental Research Institute, University of Waikato, Tauranga 3112, New Zealand; marie.magnusson@waikato.ac.nz (M.M.); christopher.glasson@waikato.ac.nz (C.R.K.G.)
[3] Centre for Marine Science and Innovation, University of New South Wales, Sydney, NSW 2052, Australia; m.majzoub@unsw.edu.au (M.E.M.); t.thomas@unsw.edu.au (T.T.)
[4] School of Biological, Earth and Environmental Sciences, University of New South Wales, Sydney, NSW 2052, Australia
[5] MACRO – The Centre for Macroalgal Resources and Biotechnology, College of Marine and Environmental Sciences, James Cook University, Townsville, QLD 4811, Australia; tine.praeger@jcu.edu.au (C.P.)
[6] School of Health and Wellbeing, University of Southern Queensland, Ipswich, QLD 4305, Australia
[*] Correspondence: lindsaybrown1952@gmail.com; Tel.: +61-433-062-123
[†] School of Health, Medical and Applied Sciences, Central Queensland University, Rockhampton, QLD 4701, Australia.
[‡] School of Science, Western Sydney University, Richmond, NSW 2753, Australia.

Citation: du Preez, R.; Magnusson, M.; Majzoub, M.E.; Thomas, T.; Praeger, C.; Glasson, C.R.K.; Panchal, S.K.; Brown, L. Brown Seaweed *Sargassum siliquosum* as an Intervention for Diet-Induced Obesity in Male Wistar Rats. *Nutrients* **2021**, *13*, 1754. https://doi.org/10.3390/nu13061754

Academic Editor: Francesca Giampieri

Received: 6 April 2021
Accepted: 19 May 2021
Published: 21 May 2021

Publisher's Note: MDPI stays neutral with regard to jurisdictional claims in published maps and institutional affiliations.

Copyright: © 2021 by the authors. Submitted for possible open access publication under the terms and conditions of the Creative Commons Attribution (CC BY) license (http://creativecommons.org/licenses/by/4.0/).

Supplementary Information

Supplementary Figure S1

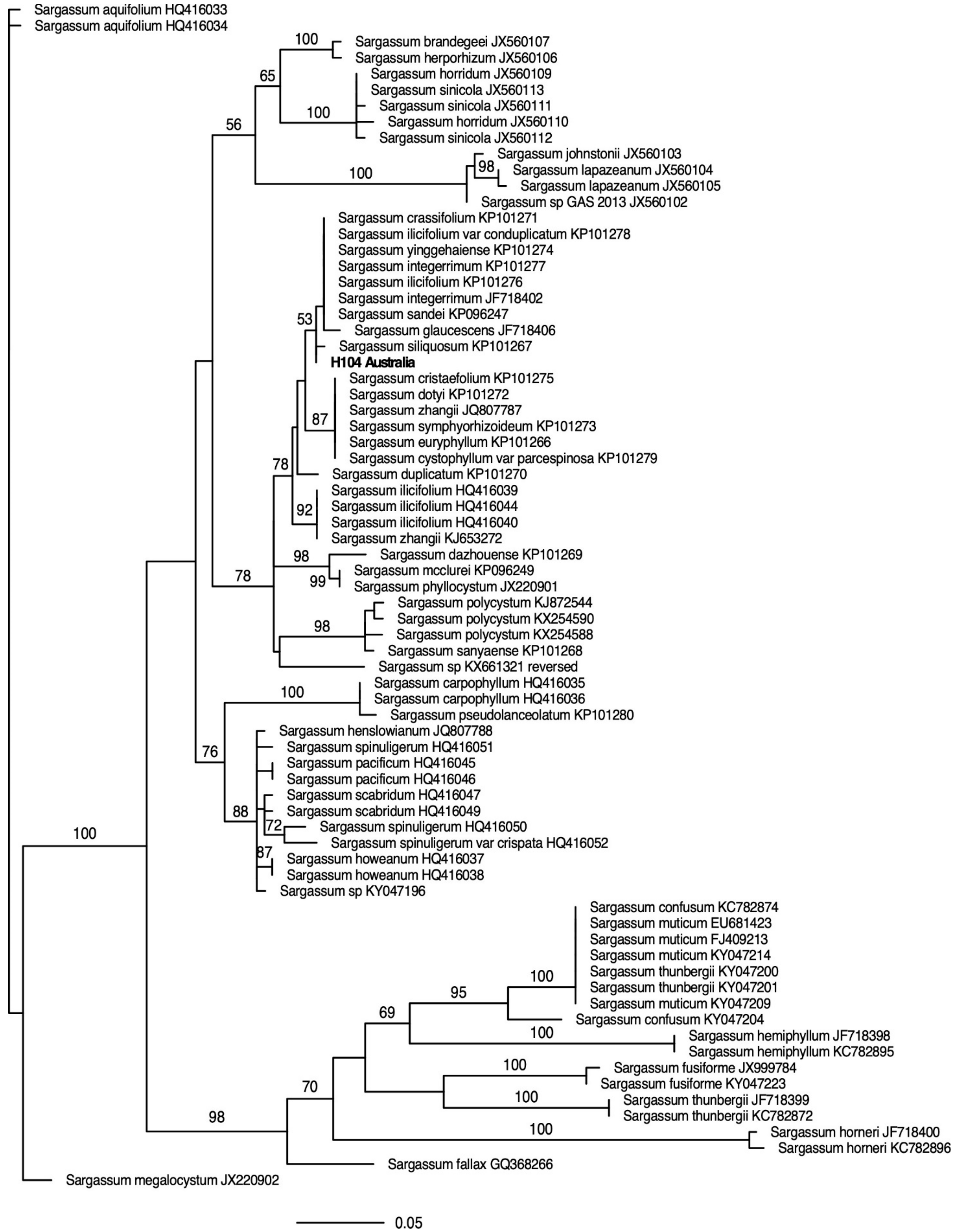

Supplementary Figure S1. Genetic barcoding of Sargassum species: Maximum likelihood tree of cytochrome oxidase I marker sequence data (full phylogenetic tree). Numbers near each node refer to bootstrap support values. Numbers accompanying the species names are GenBank accession numbers for the sequences used in the analysis. The specimens collected here are referred to as H104 Australia.

Supplementary Figure S2

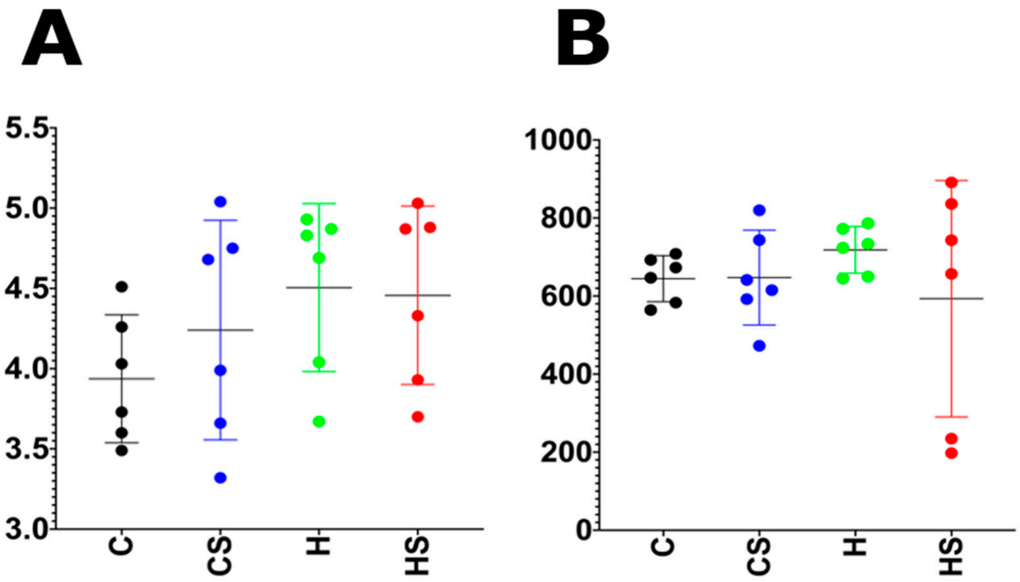

Supplementary Figure S2. (A) Shannon diversity and **(B)** richness of faecal samples. C, corn starch diet-fed rats; CS, corn starch diet-fed rats supplemented with *Sargassum siliquosum*; H, high-carbohydrate, high-fat diet-fed rats; HS, high-carbohydrate, high-fat diet-fed rats supplemented with *Sargassum siliquosum*.

Supplementary Figure S3

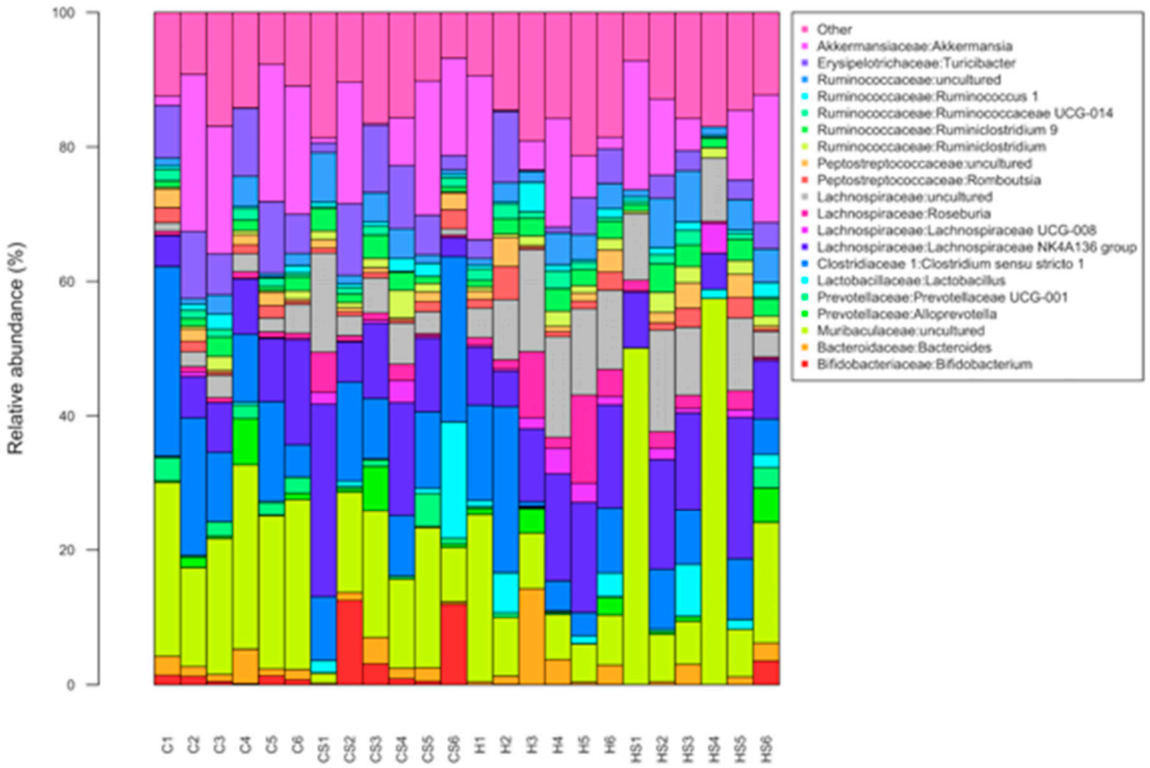

Supplementary Figure S3. Taxonomic profiles of bacterial communities of all faecal samples shown at the genus level. C, corn starch diet-fed rats; CS, corn starch diet-fed rats supplemented with *Sargassum siliquosum*; H, high-carbohydrate, high-fat diet-fed rats; HS, high-carbohydrate, high-fat diet-fed rats supplemented with *Sargassum siliquosum*.

Analysis of the bacterial community structure at the genus level showed that *Bifidobacterium* (family *Bifidobacteriaceae*), *Bacteroides* (family *Bacteroidaceae*), unclassified *Muribaculaeceae*, *Alloprevotella* (family *Prevotellaceae*), *Prevotellaceae UCG-001* (family *Prevotellaceae*), *Lactobacillus* (family *Lactobacillaceae*), *Clostridium sensu stricto 1* (family *Clostridiaceae*), *Lachnospiraceae NK4A136 group* (family *Lachnospiraceae*), *Lachnospiraceae UCG-008* (family *Lachnospiraceae*), *Roseburia* (family *Lachnospiraceae*), *uncultured Lachnospiraceae*, *Romboutsia* (family *Peptostreptococcaceae*), uncultured *Peptostreptococcaceae*, *Ruminiclostridium* (family *Ruminococcaceae*), *Ruminiclostridium 9* (family *Ruminococcaceae*), Ruminococcaceae UCG-014 (family *Ruminococcaceae*), Ruminococcus 1 (family *Ruminococcaceae*), uncultured *Ruminococcaceae*, Turicibacter (family *Erysipelotrichaceae*) and Akkermansia (family *Akkermansiaceae*) were found to be most dominant in the faecal samples.

Supplementary Tables

Supplementary Tables S1. Fatty acids and amino acids content (% of dry weight) in *Sargassum siliquosum*.

Fatty acid	% of dw	Amino acid	% of dw
C14:0	0.12 ± 0	Histidine	0.091 ± 0.001
C15:0	0.07 ± 0	Serine	0.196 ± 0.004
C16:0	0.49 ± 0.02	Arginine	0.209 ± 0.007
C16:1	0.14 ± 0	Glycine	0.214 ± 0.005
C18:0	0.02 ± 0	Aspartic acid	0.466 ± 0.013
C18:1	0.14 ± 0	Glutamic acid	0.607 ± 0.010
C18:2	0.08 ± 0.01	Threonine	0.200 ± 0.004
C18:3	0.12 ± 0.01	Alanine	0.260 ± 0.005
C20:3	0.02 ± 0	Proline	0.177 ± 0.003
C20:4	0.18 ± 0	Lysine	0.222 ± 0.007
C20:5(+C22:0)	0.08 ± 0	Tyrosine	0.120 ± 0.004
		Methionine	0.113 ± 0.006
		Valine	0.241 ± 0.006
		Isoleucine	0.210 ± 0.004
		Leucine	0.345 ± 0.008
		Phenylalanine	0.228 ± 0.005
Total fatty acids	1.47 ± 0.03	Cysteine	0.054 ± 0.002
Sum saturated fatty acids	0.7 ± 0.02	Tryptophan	0.067 ± 0.003
Sum monounsaturated fatty acids	0.28 ± 0	Sum amino acids	4.02
Sum polyunsaturated fatty acids	0.49 ± 0.02	Sum essential amino acids	1.72

Values are presented as mean ± SEM, n = 3.

Supplementary Table S2. Metals and metalloids content (mg/kg of dry weight) in *Sargassum siliquosum*.

Element	Concentration (mg. kg^{-1})
Al	1199 ± 179
As	72.9 ± 8.1
B	123 ± 4
Ba	13.5 ± 0.7
Ca	19312 ± 925
Cd	0.343 ± 0.015
Co	4.90 ± 0.29
Cr	3.30 ± 0.32
Cu	3.40 ± 0.27
Fe	774 ± 127
Hg	≤ 0.1
K	75033 ± 2785
Mg	6480 ± 265
Mn	104 ± 5
Mo	2.01 ± 0.75
Na	12226 ± 428
Ni	4.12 ± 0.21
P	581 ± 64
Pb	0.439 ± 0.156
S	9681 ± 681
Se	4.95 ± 0.33
Sr	915 ± 49
V	15.5 ± 2.6
Zn	18.9 ± 2.0

Values are presented as mean ± SEM, n = 5.

Supplementary Table S3. PERMANOVAs based on Bray-Curtis similarity measure for square-root transformed abundances of all rat faecal samples.

**PERMANOVA**						
**Source**	**df**	**SS**	**MS**	**Pseudo-F**	***P*(perm)**	**Unique perms**
Diet	1	7651.4	7651.4	4.7996	0.0003	9914
Treatment	1	2866.5	2866.5	1.7981	0.0561	9916
Diet × treatment	1	2721.5	2721.5	1.7071	0.0827	9911
Res	20	31884	1594.2			
Total	23	45123				

**PAIR-WISE TESTS**			
**Source**	**t**	**P(perm)**	**Unique perms**
C, CS	1.1632	0.079	462
C, H	2.4158	0.0023	461
C, HS	1.9267	0.0037	210
CS, H	1.8066	0.0114	462
CS, HS	1.2644	0.16	210
H, HS	1.1932	0.038	210

**PERMDISP (PAIRWISE COMPARISONS)**		
**Groups**	**t**	***P*(perm)**
C, CS	1.9455	0.0489
C, H	0.71544	0.63
C, HS	0.19444	0.91
CS, H	1.2071	0.35
CS, HS	1.349	0.28
H, HS	0.53358	0.64

P-values were calculated using 9,999 permutations under a residual model. C, corn starch diet-fed rats; CS, corn starch diet-fed rats supplemented with *Sargassum siliquosum*; H, high-carbohydrate, high-fat diet-fed rats; HS, high-carbohydrate, high-fat diet-fed rats supplemented with *Sargassum siliquosum.*

Supplementary Table S4. Summarised differential zOTU abundance.

**Global test (GLMs) by mvabund**		
Diet: $P = 0.0001$		
Treatment: $P = 0.04$		
Diet × Treatment: $P = 0.015$		
**Univariate analysis by mvabund ($P < 0.05$)**		
**Factor**	**Number of differentially abundant OTUs**	**% of total number of OTUs**
Diet	6	0.46
Treatment	1	0.08
Total (unique zOTUs affected by one or more factors)	7	0.54

Supplementary Table S5. Effects of diet on relative abundance of zOTUs.

OTU_ID	C (%)	CS (%)	H (%)	HS (%)	Phylum	Family	Genus
Zotu41	0.61	0.45	0.00	0.00	Firmicutes	*Erysipelotrichaceae*	*Allobaculum*
Zotu46	1.03	0.1	0.00	0.00	Firmicutes	*Ruminococcaceae*	*Ruminococcaceae NK4A214* group
Zotu297	0.00	0.00	0.14	0.04	Firmicutes	*Peptococcaceae*	unclassified
Zotu862	0.01	0.04	0.00	0.00	Firmicutes	*Lachnospiraceae*	unclassified
Zotu930	0.01	0.01	0.00	0.00	Firmicutes	*Ruminococcaceae*	*Ruminiclostridium 1*
Zotu1232	0.00	0.00	0.02	0.01	Firmicutes	*Ruminococcaceae*	*Ruminococcaceae UCG-014*

Differential abundance analysis was performed using *Mvabund*. C, corn starch diet-fed rats; CS, corn starch diet-fed rats supplemented with *Sargassum siliquosum*; H, high-carbohydrate, high-fat diet-fed rats; HS, high-carbohydrate, high-fat diet-fed rats supplemented with *Sargassum siliquosum.*

Supplementary Table S6. Effects of treatment on relative abundance of zOTUs.

OTU_ID	C (%)	CS (%)	H (%)	HS (%)	Phylum	Family	Genus
Zotu261	0.14	0.00	0.01	0.00	Firmicutes	*Ruminococcaceae*	*Ruminococcaceae UCG-010*

Differential abundance analysis was performed using *Mvabund*. C, corn starch diet-fed rats; CS, corn starch diet-fed rats supplemented with *Sargassum siliquosum*; H, high-carbohydrate, high-fat diet-fed rats; HS, high-carbohydrate, high-fat diet-fed rats supplemented with *Sargassum siliquosum.*
